# Dual antiplatelet therapy in patients with cirrhosis and acute myocardial infarction – A 13-year nationwide cohort study

**DOI:** 10.1371/journal.pone.0223380

**Published:** 2019-10-03

**Authors:** Victor Chien-Chia Wu, Shao-Wei Chen, An-Hsun Chou, Pei-Chi Ting, Chih-Hsiang Chang, Michael Wu, Ming-Jer Hsieh, Chao-Yung Wang, Shang-Hung Chang, Ming-Shyan Lin, Kuo-Chun Hung, I-Chang Hsieh, Pao-Hsien Chu, Cheng-Shyong Wu, Yu-Sheng Lin

**Affiliations:** 1 Division of Cardiology, Chang Gung Memorial Hospital, Linkou Medical Center, Taoyuan City, Taiwan; 2 Department of Cardiothoracic and Vascular Surgery, Chang Gung Memorial Hospital, Linkou Medical Center, Taoyuan City, Taiwan; 3 Department of Anesthesiology, Chang Gung Memorial Hospital, Linkou Medical Center, Taoyuan City, Taiwan; 4 Department of Nephrology, Chang Gung Memorial Hospital, Linkou Medical Center, Taoyuan City, Taiwan; 5 Divison of Cardiovascular Medicine, Rhode Island Hospital, Warren Alpert School of Medicine, Brown University, Providence, Rhode Island, United States America; 6 Department of Cardiology, Chang Gung Memorial Hospital, Chiayi, Taiwan; 7 Department of Gastroenterology and Hepatology, Chang Gung Memorial Hospital, Chiayi, Taiwan; East Tennessee State University, UNITED STATES

## Abstract

**Background:**

Patients with cirrhosis and acute myocardial infarction (AMI) present dilemma whether dual antiplatelet therapy (DAPT) should be used.

**Methods:**

Electronic medical records between 2001–2013 were retrieved from Taiwan National Health Insurance Research Database. Patients were excluded for missing information, age <20 years old, history of AMI, liver transplant, autoimmune disease, coagulopathy, taking DAPT 3 months before index date, follow-up <3 months, anticoagulation user, without DAPT, and events of myocardial infarction (MI), ischemic stroke, major bleeding, and heart failure within 3-month of enrollment. Primary outcomes were 1-year all-cause mortality, recurrent MI, major bleeding, and gastrointestinal bleeding.

**Results:**

A total of 150,887 patients with AMI retrieved. After exclusion criteria and propensity score-matching, 914 cirrhotic and 3,656 non-cirrhotic patients with AMI on DAPT were studied. During 1-year follow-up, there was significantly increased mortality in cirrhotic patients compared to non-cirrhotic patients (HR = 1.49, 95% CI = 1.28–1.74). There was significantly decreased recurrent MI in cirrhotic patients compared to non-cirrhotic patients (subdistribution HR [SHR] = 0.71, 95% CI = 0.54–0.92). However, non-significantly increased major bleeding (SHR = 1.23, 95% CI = 0.87–1.73) and significantly increased gastrointestinal bleeding (SHR = 1.49, 95% CI = 1.31–1.70).

**Conclusions:**

In cirrhotic patients with AMI, DAPT offers benefit with decreased recurrent MI at the expense of increased gastrointestinal bleeding.

## Introduction

Coronary artery disease (CAD) burden is found in 20%-26% of all patients with end-stage liver disease, with increased mortality up to 40% even 1 year after liver transplant [1–3]. Since cirrhotic patients are at high risk of bleeding due to the inherent complications of thrombocytopenia, coagulopathy and variceal bleeding, those who have coexisting CAD are at management dilemma. In cirrhotic patients with acute myocardial infarction (AMI), the decision to use of dual antiplatelet therapy (DAPT) therefore can be a difficult one.

Percutaneous coronary intervention (PCI) has traditionally not been an option in patients with end-stage liver disease with many debating the risk and benefits of the requisite antiplatelet use. Initially, for patients with end-stage liver disease being candidate for liver transplant, there is the mandatory management of possible CAD by PCI prior to the transplant surgery. A study with a small group of liver transplant candidates of whom the majority had significant thrombocytopenia, PCI was demonstrated feasible and safe [4]. In another study where 423 patients with cirrhosis underwent liver transplant evaluation, coronary artery stenting with antiplatelet therapy was shown to be safe in cirrhotic patients without varices [5]. However, in recent study with 148 cirrhotic patients with CAD underwent stenting, there was a significantly increased risk of gastrointestinal bleeding without affecting survival and the it remained unclear whether cardiovascular benefits of stenting with DAPT outweigh the risk of bleeding [6].

Therefore in this study, we aimed to investigate whether DAPT is an appropriate therapy in cirrhotic patients with AMI.

## Methods

### Data source

Taiwan’s National Health Institute (NHI) Program started in 1995 and provides 99.5% coverage for the 23 million residents in Taiwan. The NHI Research Database (NHIRD) provides all dates of inpatient and outpatient services, diagnosis, prescriptions, examinations, operations, and expenditures, and data are updated biannually. With over 95% of Taiwan’s population consists of Han Chinese, our study is considered of uniform ethnic background. The NHI system offers detailed follow-up information on medication, intervention, admission, outpatient clinic, and emergency visit of patients. In addition, accurate records of health reimbursement is ensured by prescription of medications and arrangement of interventions be followed by appropriate examinations and indications, otherwise false reimbursement claims results in magnified penalty. Medications for chronic illnesses were refilled at outpatient clinic with a maximum length of 3 months per the Taiwan NHI reimbursement policy. The hospital identification number of each patient was encrypted and de-identified to protect their privacy, therefore informed consent was waived for this study. The study conformed to the principles of the Helsinki Declaration and the Institutional Review Board of Chang Gung Memorial Hospital Linkou Branch approved this study.

### Study patients

By searching electronic medical records from the NHIRD between January 1, 2001 and December 31, 2013, we retrieved patients with a principal diagnosis of AMI admission. The first episode of AMI strike of each individual was selected as the index admission. We further identified patients with diagnosis of cirrhosis (two consecutive outpatient diagnoses or one inpatient diagnosis). Both the diagnostic codes of AMI and liver cirrhosis in the NHIRD have been validated against hospital electronic medical records in previous studies, with AMI and liver cirrhosis having positive predictive value of 88% and 100% respectively [7,8]. The date of discharge from the index admission was defined as the index date.

**Fig 1:**
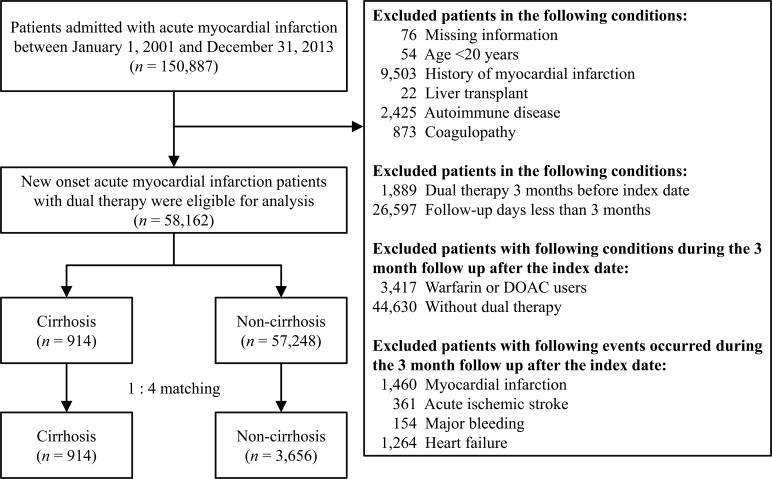
Flow chart for the inclusion the study patients.

Patients who had missing information, age <20 years old, had history of AMI, liver transplant, autoimmune disease, and coagulopathy were excluded. We also excluded patients with conditions of taking DAPT 3 months before index date and follow-up days <3 months. In addition, we excluded patients with conditions of being warfarin or direct oral anticoagulant users, without DAPT, and patients with events of myocardial infarction, ischemic stroke, major bleeding, and heart failure within 3 months of the index date. The remaining were patients with new onset of AMI with DAPT. These patients were separated into cirrhotic patients and non-cirrhotic patients. (**Fig 1**). In addition, a separated analysis for cirrhotic patients who were prescribed DAPT group or single antiplatelet therapy (SAPT) after AMI. In our study, DAPT means prescribing aspirin plus clopidogrel while SAPT means prescribing aspirin alone or clopidogrel alone.

### Covariate and study outcomes

Disease was detected using International Classification of Diseases, 9th Revision, Clinical Modification (ICD-9-CM) codes. Covariates included gender, age, socioeconomic factor (monthly income and urbanization level), clinical medical history of hypertension, diabetes mellitus, hyperlipidemia, heart failure, peripheral arterial disease, atrial fibrillation, history of stroke, major bleeding, gastrointestinal bleeding, chronic kidney disease, end-stage renal disease, malignancy, and Charlson comorbidity score. The coronary intervention in prior admissions and at the index admission was also recorded. The information of post MI medications were also extracted. Most comorbidities were defined as having two outpatient diagnoses or one inpatient diagnosis in the previous year and also some cirrhosis-related or AMI-related complications were also defined according to previous study [9] or the diagnosis of ICD-9-CM. Some severe comorbidities were detected in inpatient diagnoses only, including heart failure, stroke, major bleeding, and gastrointestinal bleeding which can be tracked to Jan 1 1997. Usage of medication was retrieved based on claim data within 3 months of the index date.

Outcomes of primary interest included all-cause mortality, recurrent myocardial infarction (MI), major bleeding [10], and gastrointestinal bleeding. All-cause mortality was defined by a withdrawal from the national health insurance [11]. The recurrent MI was defined as a principal diagnosis in an admission setting and the diagnostic code was validated in a previous study [12]. Bleeding events were detected in an admission setting as well and the definition was widely reported in previous NHIRD studies [13,14]. Each patient was followed until the day of outcome occurrence, death or December 31, 2013, whichever came first.

### Statistical analysis

To reach a comparability between the cirrhotic and non-cirrhotic groups, we conducted a propensity score matching. Each cirrhotic patient was matched with 4 non-cirrhotic patients. The propensity score was the predicted probability to be in the cirrhotic group derived from a multivariable logistic regression given value of covariates. The covariates were the aforementioned covariates in which the follow up year was replaced with the index date. Noticeably, the CCI score was not included in the calculation of propensity score because of liver disease was a component of CCI in which 1 point and 3 points were assigned to mild and severe liver diseases respectively. The matching was processed using a greedy nearest neighbor algorithm with a caliper of 0.2 times the standard deviation of the logit of propensity score and without replacement and with random matching order. The quality of matching was verified using the absolute value of standardized mean difference (SMD) which a value less than 0.1 was considered to have negligible difference between groups.

We compared the risk of all-cause mortality between groups using the Cox proportional hazard model. The risk of other time to event outcomes (recurrent MI and bleeding events) between groups was compared using the Fine and Gray subdistribution hazard model which considered death as a competing risk. Matching pairs were stratified in both Cox and Fine and Gray models. Unadjusted cumulative event rate of all-cause mortality was calculated and plotted. The cumulative incidence of recurrent MI and bleeding events was generated and plotted using subdistribution cumulative incidence function. The study group (cirrhosis vs. non-cirrhosis) was the only explanatory variable in the survival analyses.

We further performed a separate analysis to compare DAPT with SAPT in the cirrhotic patients. There were relatively small sample size in these two groups and substantially differences in the baseline characteristics (**S1 Table**) between groups, it would be infeasible to conduct propensity score matching. Therefore, we calculated a propensity score which was the predicted probability to be the DAPT group relative to be the SAPT group using the same covariates as the primary analysis. This propensity score was adjusted in the survival analyses when comparing the two groups [15]. A two-sided *P* value < 0.05 was considered to be statistically significant and no adjustment of multiple testing (multiplicity) was made in this study. All statistical analyses were performed using SAS version 9.4 (SAS Institute, Cary, NC), including procedure of ‘*psmatch*’ for propensity score matching, ‘*phreg*’ for survival analysis, and the macro of ‘*%cif*’ for cumulative incidence function.

## Results

### Study population

There were 150,887 patients admitted with a principal diagnosis of AMI during 2001 and 2013 in the NHIRD. After applying exclusion criteria, there remained 58,162 patients with new onset of AMI on DAPT and were separated into 914 (1.6%) cirrhotic patients and 57,248 (98.4%) non-cirrhotic patients. After conducting a 1:4 propensity score matching, there were 914 patients with cirrhosis and 3,656 patients without cirrhosis (**Fig 1**). The distribution of baseline characteristics, comorbidities, hospital level, coronary intervention, medications, and follow up duration were similar between the two groups (right panel in **Table 1**). Besides, the cirrhosis related clinical characteristics of these 914 cirrhotic patients were listed in **Table 2**.

**Table 1:** Clinical characteristics of study population before and after propensity score matching.

	Before matching			After matching		
Variable	Cirrhosis (*n* = 914)	Non-cirrhosis (*n* = 57,248)	SMD	Cirrhosis (*n* = 914)	Non-cirrhosis (*n* = 3,656)	SMD
Characteristics						
Age, years	66.0±12.3	62.1±13.4	0.298	66.0±12.3	65.8±12.5	0.012
Age ≥ 65	469 (51.3)	23,866 (41.7)	0.194	469 (51.3)	1,835 (50.2)	0.022
Male gender	754 (82.5)	45,753 (79.9)	0.066	754 (82.5)	3,047 (83.3)	-0.023
Monthly income, NT$						
Low (0–17880)	334 (36.5)	18,766 (32.8)	0.079	334 (36.5)	1,290 (35.3)	0.026
Medium (17881–22800)	323 (35.3)	17,968 (31.4)	0.084	323 (35.3)	1,337 (36.6)	-0.026
High (> 22800)	257 (28.1)	20,514 (35.8)	-0.166	257 (28.1)	1,029 (28.1)	-0.001
Urbanization level						
Rural	151 (16.5)	6,580 (11.5)	0.145	151 (16.5)	613 (16.8)	-0.007
Town	273 (29.9)	16,588 (29.0)	0.020	273 (29.9)	1,102 (30.1)	-0.006
Urban	272 (29.8)	18,229 (31.8)	-0.045	272 (29.8)	1,086 (29.7)	0.001
Metropolis	218 (23.9)	15,851 (27.7)	-0.088	218 (23.9)	855 (23.4)	0.011
Comorbidity						
Hypertension	645 (70.6)	35,334 (61.7)	0.188	645 (70.6)	2,592 (70.9)	-0.007
Diabetes mellitus	443 (48.5)	20,230 (35.3)	0.269	443 (48.5)	1,762 (48.2)	0.005
Hyperlipidemia	334 (36.5)	28,189 (49.2)	-0.259	334 (36.5)	1,348 (36.9)	-0.007
Heart failure	97 (10.6)	2,317 (4.0)	0.254	97 (10.6)	387 (10.6)	0.001
Peripheral arterial disease	48 (5.3)	1,543 (2.7)	0.131	48 (5.3)	186 (5.1)	0.007
Atrial fibrillation	70 (7.7)	2,566 (4.5)	0.133	70 (7.7)	261 (7.1)	0.020
Old stroke	176 (19.3)	5,541 (9.7)	0.275	176 (19.3)	723 (19.8)	-0.013
Old major bleeding	95 (10.4)	2,200 (3.8)	0.257	95 (10.4)	376 (10.3)	0.004
Old gastrointestinal bleeding	308 (33.7)	6,047 (10.6)	0.580	308 (33.7)	1,228 (33.6)	0.002
Chronic kidney disease	233 (25.5)	7,466 (13.0)	0.320	233 (25.5)	924 (25.3)	0.005
ESRD (dialysis)	73 (8.0)	1,419 (2.5)	0.249	73 (8.0)	281 (7.7)	0.011
Malignancy	128 (14.0)	1,879 (3.3)	0.389	128 (14.0)	496 (13.6)	0.013
CCI total score (unmatched)	4.0±2.3	2.3±1.7	0.819	4.0±2.3	3.3±2.3	0.277
Hospital level						
Medical center (teaching hospital)	416 (45.5)	28,133 (49.1)	-0.073	416 (45.5)	1,697 (46.4)	-0.018
Regional / district hospital	498 (54.5)	29,115 (50.9)	0.073	498 (54.5)	1,959 (53.6)	0.018
Prior CABG	12 (1.3)	419 (0.7)	0.058	12 (1.3)	55 (1.5)	-0.016
Prior PCI	61 (6.7)	2,217 (3.9)	0.126	61 (6.7)	246 (6.7)	-0.002
Coronary intervention at the index admission						
CABG	2 (0.2)	257 (0.4)	-0.040	2 (0.2)	6 (0.2)	0.013
PCI	709 (77.6)	46,568 (81.3)	-0.093	709 (77.6)	2,829 (77.4)	0.005
BMS	425 (59.9)	27,584 (59.2)	0.014	425 (59.9)	1,704 (60.2)	-0.006
DES	151 (21.3)	10,346 (22.2)	-0.022	151 (21.3)	567 (20.0)	0.031
Post MI medications						
ACEI / ARB	606 (66.3)	39,517 (69.0)	-0.058	606 (66.3)	2,372 (64.9)	0.030
Beta blocker	534 (58.4)	37,083 (64.8)	-0.131	534 (58.4)	2,117 (57.9)	0.011
DCCB	160 (17.5)	7,908 (13.8)	0.102	160 (17.5)	620 (17.0)	0.014
Alpha blocker	37 (4.0)	1,453 (2.5)	0.085	37 (4.0)	151 (4.1)	-0.004
Nitrates	175 (19.1)	10,124 (17.7)	0.038	175 (19.1)	720 (19.7)	-0.014
Diuretics (Loop diuretics, Spironolactone, Thiazide)	218 (23.9)	10,484 (18.3)	0.136	218 (23.9)	857 (23.4)	0.010
OHA	290 (31.7)	15,526 (27.1)	0.101	290 (31.7)	1,183 (32.4)	-0.013
Insulin	117 (12.8)	2,586 (4.5)	0.298	117 (12.8)	455 (12.4)	0.011
Statin	442 (48.4)	34,682 (60.6)	-0.247	442 (48.4)	1,792 (49.0)	-0.013
Digoxin	20 (2.2)	1,487 (2.6)	-0.027	20 (2.2)	77 (2.1)	0.006
PPI	76 (8.3)	2,067 (3.6)	0.200	76 (8.3)	295 (8.1)	0.009
Follow-up (years) (unmatched)	3.4±2.6	4.3±3.0	-0.337	3.4±2.6	3.6±2.7	-0.079
Propensity score	0.039±0.047	0.015±0.019	0.669	0.039±0.047	0.039±0.045	0.009

SMD, standardized mean difference; ESRD, end stage renal disease; CCI, Charlson Comorbidity Index; PCI, percutaneous coronary intervention; CABG, coronary artery bypass grafting; BMS, bare-metal stent; DES, drug-eluting stent; ACEI, angiotensin-converting enzyme inhibitors, ARB, angiotensin receptor blockers; DCCB, dihydropyridine calcium channel blockers; OHA, oral hypoglycemic agent; PPI, proton-pump inhibitor.

**Table 2:** Liver cirrhosis related clinical characteristics of the patients.

Variable	Cirrhosis (*n* = 914)
Alcoholic cirrhosis	118 (12.9)
Virus hepatitis, HBV	191 (20.9)
Virus hepatitis, HCV	167 (18.3)
Old gastrointestinal bleeding	308 (33.7)
Old major bleeding	95 (10.4)
Complication of cirrhosis	
Hepatic encephalopathy	20 (2.2)
Ascites and related complication	81 (8.9)
Esophageal varices bleeding	26 (2.8)
Admission for albumin infusion (hypoalbuminemia)	74 (8.1)
Catastrophic illness certificate	
No	898 (98.2)
Yes	16 (1.8)

### Cirrhotic versus non-cirrhotic

**Fig 2:**
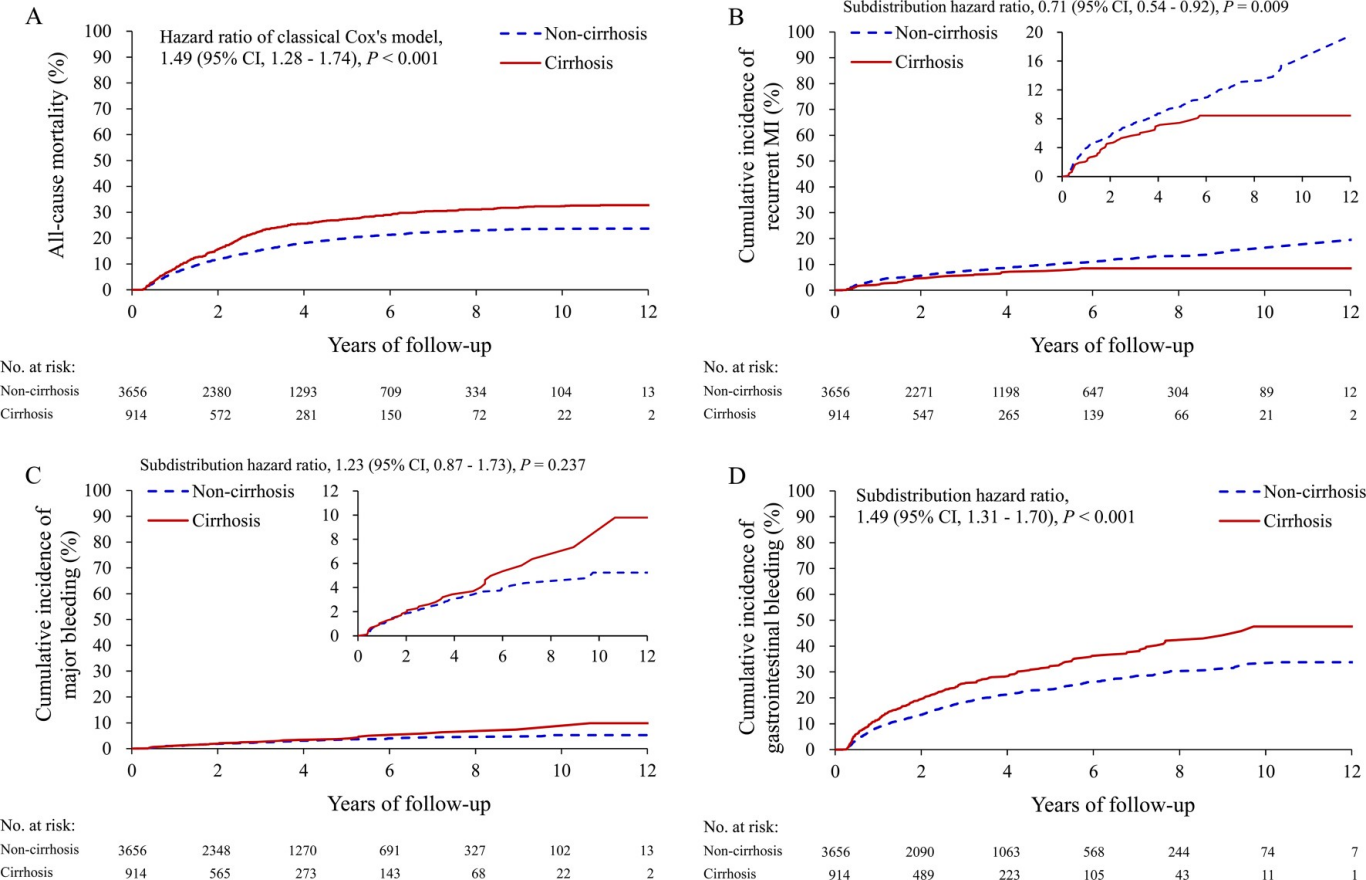
Unadjusted cumulative event rate of all-cause mortality (A) and cumulative incidence of recurrent MI (B), major bleeding (C) and gastrointestinal bleeding (D) in the cirrhotic and non-cirrhotic patients with acute MI. MI, myocardial infarction.

We first compared the risk of primary outcomes in cirrhotic and non-cirrhotic patients with AMI using DAPT. During the 1 year follow up, there was a significantly increased mortality rate in the cirrhotic patients compared to the non-cirrhotic patients (32.7% vs. 23.7%; hazard ratio [HR] 1.49, 95% confidence interval [CI] 1.28–1.74) (**Fig 2A**). There was significantly decreased recurrent MI in the cirrhotic patients compared to the non-cirrhotic patients (6.0% vs. 8.7%; subdistribution HR [SHR] 0.71, 95% CI 0.54–0.92) (**Fig 2B**). However, there were non-significantly increased major bleeding (3.7% vs. 2.9%; SHR 1.23, 95% CI 0.87–1.73) (**Fig 2C**) but significantly increased gastrointestinal bleeding (28.0% vs. 20.2%; SHR 1.49, 95% CI 1.31–1.70) (**Fig 2D**).

### Single versus dual antiplatelet therapy

In the cirrhotic patients, we further compared outcomes of patients prescribed with DAPT and SAPT, as bleeding is the main concern regarding this special population. **S1 Table** shows the baseline clinical characteristics of patients using SAPT and DAPT. Compared to SAPT, DAPT lead to significantly decreased all-cause mortality (32.7% vs. 58.5%; HR 0.77, 95% CI 0.63–0.94) (**S1A Fig**) and non-significantly decreased recurrent MI (6.0% vs. 9.2%; SHR 0.79, 95% CI 0.49–1.27) (**S1B Fig**). For bleeding conditions, compared to SAPT, DAPT lead to similar risks of major bleeding (3.7% vs. 5.5%; SHR 1.08, 95% CI 0.57–2.05) (**S1C Fig**) and gastrointestinal bleeding (28.0% vs. 37.8%; SHR 1.05, 95% CI 0.82–1.35) (**S1D Fig**). Finally, an analysis for the different antiplatelet therapy in cirrhotic patients prescribed with SAPT was performed and it showed non-significant in individual outcome between them. **(S2 Table).**

## Discussion

Our study has the following findings. (1) This is the largest study to date to directly compare the clinical outcomes of DAPT in cirrhotic patients with AMI and non-cirrhotic patients with AMI, which showed DAPT had significant benefit in preventing recurrent MI in cirrhotic patients with AMI compared to non-cirrhotic patient with AMI (2) This is the first study to compare the clinical outcomes DAPT and SAPT in cirrhotic patients with AMI, which showed similar protection of DAPT and SAPT in recurrent MI, major bleeding, and gastrointestinal bleeding in physician directed treatment.

In patients with liver cirrhosis, bleeding diathesis is an important consideration when antiplatelet is a necessary therapy whether it is CAD, cerebrovascular disease, or peripheral vascular disease. In these patients who have CAD undergoing PCI requiring DAPT, the situation becomes a treatment dilemma. There is currently no guideline recommendation, and only limited evidences exist regarding the effectiveness and safety on the use of antiplatelet in cirrhotic patients from previously published studies [4–6].

In this study, we enrolled patients with AMI and separated into cirrhotic and non-cirrhotic patients with propensity score matching performed between groups. During the one year follow up, we showed that the cirrhotic patients with AMI had decreased recurrent AMI using DAPT compared to non-cirrhotic patients using DAPT. Our finding is important because in this situation, we are confirmed the benefits of DAPT even more prominent in cirrhotic patients. Although use of DAPT was encountered with more bleeding events, mainly gastrointestinal bleeding, there was a non-significant increase of major bleeding, suggesting it may related to old gastrointestinal bleeding (33.7%) but not variceal bleeding due to its small percentage (2.8%) (**Table 2**). The all-cause mortality was still higher in cirrhotic patients with AMI, although if not DAPT prescribed, the overall mortality rate may be still higher.

To investigate whether DAPT or SAPT should be recommended in cirrhotic patients with AMI, we did secondary analysis of primary outcomes in this special population. As seen in **S1 Table**, the baseline characteristics showed patients who were prescribed with SAPT had higher percentage of heart failure, old gastrointestinal bleeding suggest poorer underlying diseases. Hence, patients who were prescribed with SAPT also had lower percentage of ACEi/ARB, beta blockers, nitrates, and statin, and a higher percentage of digoxin and proton pump inhibitor given, which in turn reflected the poorer underlying comorbidities. Under these conditions, use of DAPT showed no difference in terms of recurrent MI, major bleeding, and gastrointestinal bleeding compared to use of SAPT in these cirrhotic patients that may be seen with different coexisting diseases. Under better coexisting conditions, cirrhotic patients with AMI could be prescribed with the standard DAPT as in non-cirrhotic patients with AMI. Together the results however, suggested that in patients with cirrhosis, whether DAPT or SAPT should be used is justified by the physician’s discretion.

In summary, this is the largest study to directly compare selective DAPT in cirrhotic and non-cirrhotic patients for the clinical outcomes. DAPT offers benefits in recurrent MI while suffers from increased risks of gastrointestinal bleeding. SAPT may be judged appropriately and prescribed according to clinical situations in patients with cirrhosis.

## Limitations

There are several limitations in epidemiologic data from NHIRD. First, using ICD-9-CM codes for patient screening may miss some cases for conditions not coded correctly. However, ICD-9-CM codes against hospital electronic medical records (EMR) have been performed in the validation studies for NHIRD, the ICD codes have as sensitivity up to 99% for positive predictive value against the gold standard EMR. Second, due to the limitation of NHIRD where laboratory results and clinical evaluation were unavailable, the traditional risk stratification using Child-Pugh criteria in patients with liver cirrhosis could not be performed. However, we substituted Child-Pugh score with surrogate markers for cirrhosis severity such as the requirement for fresh frozen plasma and albumin transfusion to indicate the patients’ coagulopathy and hypoalbuminemia, and application of which have been used in our previous works [16,17]. Thus the results of our statistical analysis may have partially ruled out the confounding due to the lack of Child-Pugh score when comparing outcomes between the SAPT and DAPT patients groups. Third, we did not analyze whether major bleeding contributed to increased all-cause mortality during DAPT use in cirrhotic patients. Forth, hospital data suffer from selection bias. Since the data we can analyze are derived from those patients who required the services from the hospitals and clinics, and those who adhere to physicians’ orders, therefore this is a limitation of the study. On the other hand, Taiwan’s National Health Institute (NHI) Program started in 1995 and provides 99.5% coverage for the 23 million residents in Taiwan. Therefore, this is the most comprehensive real-world data that the researchers can access. Fifth, the adherence cannot be assessed in this study However, we assumed “good adherence” wound be in our study base on patients who had severe disease such as acute myocardial infarction would not easily change their medication uses and would adhere to physicians’ order. In addition, we included patients who had been taking antiplatelets for at least 3 months, and the treatment of antiplatelet up to 9 months is reimbursed by the NHI. Sixth, we did not compare clopidogrel versus ticagrelor in our study and further studies maybe required for risk and benefit of these antiplatelet agents. Last, since the current study derived from the Han Chinese in Taiwan, applications of our results to other population require further studies.

## Conclusions

In cirrhotic patients with AMI, DAPT offers benefit with decreased recurrent MI at the expense of increased gastrointestinal bleeding. In cirrhotic patients with worse comorbidities, SAPT may be considered. In addition, further randomized controlled trials were needed to further evaluate the benefit of DAPT (short term vs long term) therapy in cirrhotic patients.

## Supporting information

S1 Fig Unadjusted cumulative event rate of all-cause mortality (A) and cumulative incidence of recurrent MI (B), major bleeding (C) and gastrointestinal bleeding (D) in the DAPT and SAPT users in the cirrhotic patients with acute MI. MI, myocardial infarction; DAPT, dual antiplatelet therapy; SAPT, single antiplatelet therapy.
(TIF)

S1 Table Clinical characteristics of dual and single antiplatelet therapy populations. (DOCX)

S2 Table Clinical outcomes between cirrhotic patients prescribed with clopidogrel alone or aspirin alone. (DOCX)
