# Supplementary figures and images for: Dual antiplatelet therapy in patients with cirrhosis and acute myocardial infarction – A 13-year nationwide cohort study
Source: PLoS One. 2019 Oct 3;14(10):e0223380. doi: 10.1371/journal.pone.0223380 (PMC6776333; doi:10.1371/journal.pone.0223380)

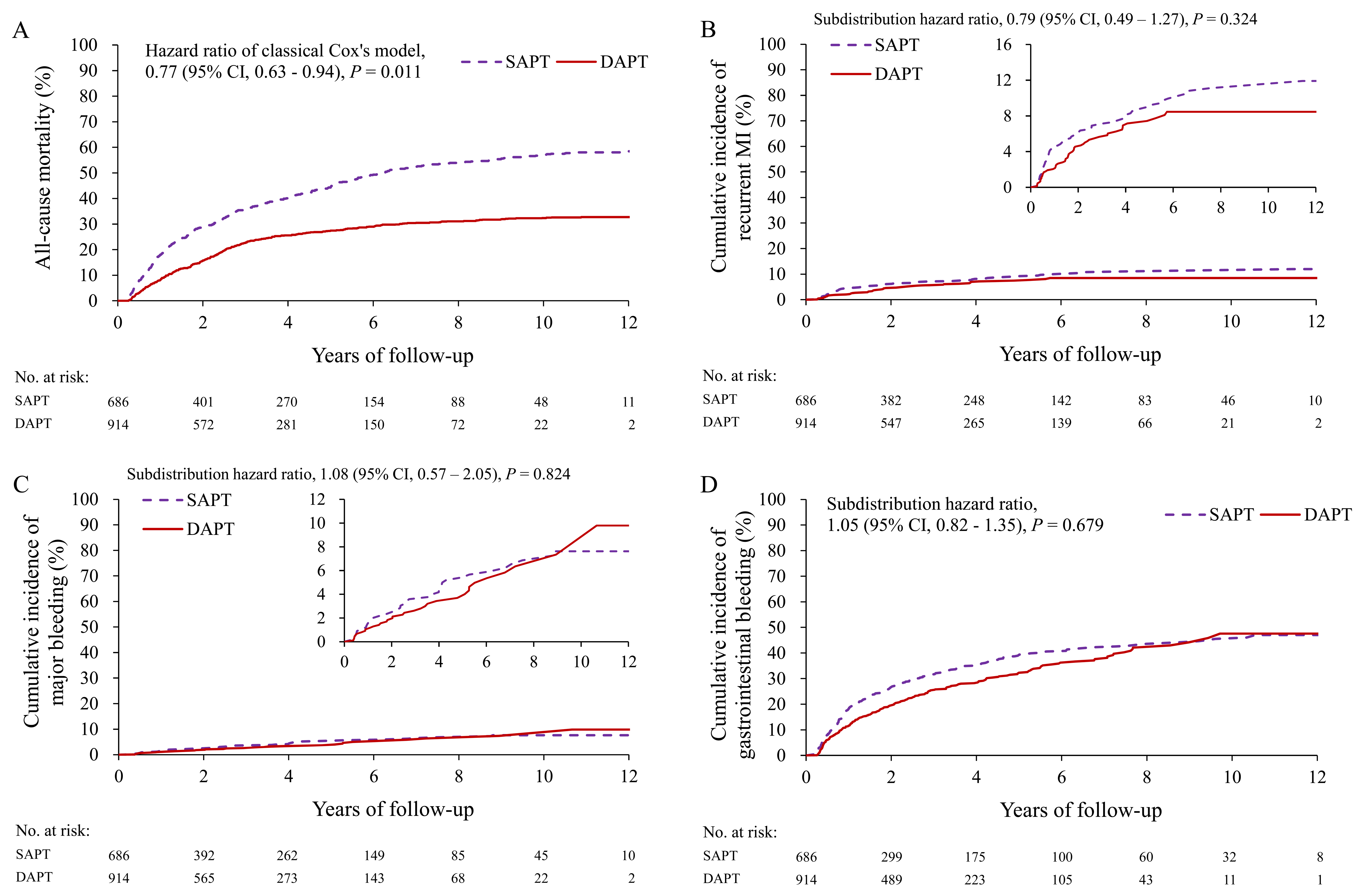

Supplement: S1 Fig — Unadjusted cumulative event rate of all-cause mortality (A) and cumulative incidence of recurrent MI (B), major bleeding (C) and gastrointestinal bleeding (D) in the DAPT and SAPT users in the cirrhotic patients with acute MI. MI, myocardial infarction; DAPT, dual antiplatelet therapy; SAPT, single antiplatelet therapy. (TIF) [file pone.0223380.s001.tif]
