# Supplementary material for: Dual antiplatelet therapy in patients with cirrhosis and acute myocardial infarction – A 13-year nationwide cohort study
Source: PLoS One. 2019 Oct 3;14(10):e0223380. doi: 10.1371/journal.pone.0223380 (PMC6776333; doi:10.1371/journal.pone.0223380)
Supplement: S1 Table — (DOCX) [file pone.0223380.s002.docx]

**S1 Table**. Clinical characteristics of dual and single antiplatelet therapy populations

| Variable | Dual  (*n* = 914) | Single  (*n* = 686) | *P* value |
| --- | --- | --- | --- |
| Characteristics |  |  |  |
| Age, years | 66.0±12.3 | 68.1±12.4 | 0.001 |
| Age ≥ 65 | 469 (51.3) | 420 (61.2) | <0.001 |
| Male gender | 754 (82.5) | 494 (72.0) | <0.001 |
| Monthly income, NT$ |  |  | 0.006 |
| Low (0–17880) | 334 (36.5) | 272 (39.7) |  |
| Medium (17881–22800) | 323 (35.3) | 269 (39.2) |  |
| High (> 22800) | 257 (28.1) | 145 (21.1) |  |
| Urbanization level |  |  | 0.392 |
| Rural | 151 (16.5) | 124 (18.1) |  |
| Town | 273 (29.9) | 224 (32.7) |  |
| Urban | 272 (29.8) | 190 (27.7) |  |
| Metropolis | 218 (23.9) | 148 (21.6) |  |
| Comorbidity |  |  |  |
| Hypertension | 645 (70.6) | 500 (72.9) | 0.309 |
| Diabetes mellitus | 443 (48.5) | 330 (48.1) | 0.886 |
| Hyperlipidemia | 334 (36.5) | 163 (23.8) | <0.001 |
| Heart failure | 97 (10.6) | 131 (19.1) | <0.001 |
| Peripheral arterial disease | 48 (5.3) | 27 (3.9) | 0.218 |
| Atrial fibrillation | 70 (7.7) | 76 (11.1) | 0.019 |
| Old stroke | 176 (19.3) | 140 (20.4) | 0.567 |
| Old major bleeding | 95 (10.4) | 102 (14.9) | 0.007 |
| Old gastrointestinal bleeding | 308 (33.7) | 312 (45.5) | <0.001 |
| Chronic kidney disease | 233 (25.5) | 205 (29.9) | 0.051 |
| ESRD (dialysis) | 73 (8.0) | 49 (7.1) | 0.529 |
| Malignancy | 128 (14.0) | 86 (12.5) | 0.393 |
| CCI total score (un-estimated for PS) | 4.0±2.3 | 4.7±2.4 | <0.001 |
| Hospital level |  |  | 0.006 |
| Medical center (teaching hospital) | 416 (45.5) | 265 (38.6) |  |
| Regional / district hospital | 498 (54.5) | 421 (61.4) |  |
| Prior CABG | 12 (1.3) | 7 (1.0) | 0.593 |
| Prior PCI | 61 (6.7) | 41 (6.0) | 0.572 |
| Coronary intervention at the index admission |  |  |  |
| CABG | 2 (0.2) | 42 (6.1) | <0.001 |
| PCI | 709 (77.6) | 183 (26.7) | <0.001 |
| BMS | 425 (59.9) | 79 (43.2) | <0.001 |
| DES | 151 (21.3) | 19 (10.4) | 0.001 |
| Post MI medications |  |  |  |
| ACEI / ARB | 606 (66.3) | 340 (49.6) | <0.001 |
| Beta blocker | 534 (58.4) | 284 (41.4) | <0.001 |
| DCCB | 160 (17.5) | 145 (21.1) | 0.067 |
| Alpha blocker | 37 (4.0) | 41 (6.0) | 0.076 |
| Nitrates | 175 (19.1) | 171 (24.9) | 0.005 |
| Diuretics (Loop diuretics, Spironolactone, Thiazide) | 218 (23.9) | 259 (37.8) | <0.001 |
| OHA | 290 (31.7) | 197 (28.7) | 0.195 |
| Insulin | 117 (12.8) | 81 (11.8) | 0.550 |
| Statin | 442 (48.4) | 160 (23.3) | <0.001 |
| Digoxin | 20 (2.2) | 50 (7.3) | <0.001 |
| PPI | 76 (8.3) | 91 (13.3) | 0.001 |
| Follow-up (years) (un-estimated for PS) | 3.4±2.6 | 3.8±3.2 | 0.008 |
| Liver cirrhosis related |  |  |  |
| Alcoholic cirrhosis | 118 (12.9) | 102 (14.9) | 0.260 |
| Virus hepatitis, HBV | 191 (20.9) | 119 (17.3) | 0.075 |
| Virus hepatitis, HCV | 167 (18.3) | 138 (20.1) | 0.352 |
| Complication of cirrhosis |  |  |  |
| Hepatic encephalopathy | 20 (2.2) | 38 (5.5) | <0.001 |
| Ascites and related complication | 81 (8.9) | 78 (11.4) | 0.097 |
| Esophageal varices bleeding | 26 (2.8) | 40 (5.8) | 0.003 |
| Admission for albumin infusion (hypoalbuminemia) | 74 (8.1) | 56 (8.2) | 0.961 |
| Catastrophic illness certificate |  |  | 0.007 |
| No | 898 (98.2) | 659 (96.1) |  |
| Yes | 16 (1.8) | 27 (3.9) |  |
| Propensity score (PS) | 0.749±0.224 | 0.335±0.267 | <0.001 |

ESRD, end stage renal disease; CCI, Charlson Comorbidity Index; PCI, percutaneous coronary intervention; CABG, coronary artery bypass grafting; BMS, bare-metal stent; DES, drug-eluting stent; ACEI, angiotensin-converting enzyme inhibitors, ARB, angiotensin receptor blockers; DCCB, dihydropyridine calcium channel blockers.
