# Supplementary material for: Dual antiplatelet therapy in patients with cirrhosis and acute myocardial infarction – A 13-year nationwide cohort study
Source: PLoS One. 2019 Oct 3;14(10):e0223380. doi: 10.1371/journal.pone.0223380 (PMC6776333; doi:10.1371/journal.pone.0223380)
Supplement: S2 Table — (DOCX) [file pone.0223380.s003.docx]

**S2 Table**. Clinical outcomes between cirrhotic patients prescribed with clopidogrel alone or aspirin alone

|  | Clopidogrel  (*n* = 268) | Aspirin  (*n* = 418) |  | Clopidogrel *vs*. Aspirin | |  | Clopidogrel *vs*. Aspirin† | |
| --- | --- | --- | --- | --- | --- | --- | --- | --- |
| Outcomes |  |  |  | HR (95% CI) # | *P*-value |  | aHR (95% CI) # | *P*-value |
| All-cause mortality | 161 (60.1) | 240 (57.4) |  | 1.65 (1.34, 2.02) | <0.001 |  | 1.23 (0.94, 1.60) | 0.127 |
| Recurrent myocardial infarction | 21 (7.8) | 42 (10.0) |  | 0.85 (0.51, 1.44) | 0.554 |  | 0.80 (0.43, 1.48) | 0.473 |
| Major bleeding | 13 (4.9) | 25 (6.0) |  | 0.93 (0.48, 1.79) | 0.820 |  | 1.02 (0.47, 2.21) | 0.958 |
| Gastrointestinal bleeding | 111 (41.4) | 148 (35.4) |  | 1.48 (1.16, 1.89) | 0.002 |  | 1.29 (0.96, 1.74) | 0.088 |

HR, hazard ratio; CI, confidence interval;

† Propensity score as a coariate adjustment;

# Estimated using Fine and Gray (1999) subdistribution hazard model which considered death as a competing risk. The results of all-cause mortality was derived from Cox proportional hazard model.
